# Supplementary material for: Chronic anti-coagulation therapy reduced mortality in patients with high cardiovascular risk early in COVID-19 pandemic
Source: Thromb J. 2023 Jan 30;21:14. doi: 10.1186/s12959-023-00460-z (PMC9885912; doi:10.1186/s12959-023-00460-z)
Supplement: Supplementary file 1 — Additional file 1: eTable 1. Unadjusted logistic regression model of propensity matched analysis of COVID-19 associated MOSC between AC and Control groups. [file 12959_2023_460_MOESM1_ESM.docx]

**Supplemental data**

**eTable 1. Unadjusted logistic regression model of propensity matched analysis of COVID-19 associated MOSC between AC and Control groups**

| **Outcome** | **OR** | **95% Confidence Interval** | **P-value** |
| --- | --- | --- | --- |
| Stroke | 3.02 | 2.33 – 3.92 | <0.0001 |
| Limb ischemia | 3.05 | 0.95 – 9.77 | 0.0609 |
| GI bleeding | 12.47 | 5.12 – 30.36 | <0.0001 |
| ICU death | 2.13 | 1.52 – 2.80 | <0.0001 |
| In-hospital death | 2.33 | 1.65 – 3.30 | <0.0001 |
